# Interleukin-6 modulates endoplasmic reticulum stress signaling and mitochondrial protein complexes in the kidney following acute exhaustive exercise

**DOI:** 10.1016/j.cstres.2025.100111

**Published:** 2025-09-02

**Authors:** Adelino S.R. da Silva, Caroline M. da Luz, Bruno B. Marafon, Maria Eduarda A. Tavares, Ivo Vieira de.S. Neto, Ruither O. Gomes Carolino, Driele C. da Silva Ferreira, Julia T. Marinho, Giovana R. Teixeira, Dennys E. Cintra, José R. Pauli, Eduardo R. Ropelle, Ellen C. de Freitas, Ana P. Pinto

**Affiliations:** 1 School of Physical Education and Sport of Ribeirão Preto, University of São Paulo (USP), Ribeirão Preto, São Paulo 14040-900, Brazil; 2 Postgraduate Program in Rehabilitation and Functional Performance, Ribeirão Preto Medical School, University of São Paulo (USP), Ribeirão Preto, São Paulo 14049-900, Brazil; 3 Department of Physical Education, State University of São Paulo (UNESP), Presidente Prudente, São Paulo 19060-900, Brazil; 4 Multicentric Program of Postgraduate in Physiological Sciences, São Paulo State University (UNESP), School of Dentistry of Araçatuba, Araçatuba, São Paulo 16015-050, Brazil; 5 Laboratory of Nutritional Genomics, School of Applied Sciences, University of Campinas (UNICAMP), Limeira, São Paulo 13484-350, Brazil; 6 Obesity and Comorbidities Research Center, UNICAMP, Campinas, São Paulo 13083-864, Brazil; 7 Laboratory of Molecular Biology of Exercise (LaBMEx), School of Applied Sciences, University of Campinas (UNICAMP), Limeira, São Paulo 13484-350, Brazil; 8 Department of Health Sciences, Ribeirão Preto Medical School, University of São Paulo (USP), Ribeirão Preto, São Paulo 14049-900, Brazil

**Keywords:** Kidney, Mast cells, Cytokines, UPR, Running exercise, IL-6

## Abstract

Endoplasmic Reticulum (ER) homeostasis is closely regulated by an adaptive signaling network identified as the unfolded protein response (UPR), which is tightly related to the inflammatory pathway. However, physical exercise increases plasma concentrations of interleukin-6 (IL-6), which exhibits both pro- and anti-inflammatory properties that mediate ER function and mitochondrial metabolism, making its investigation relevant in physiological and pathological contexts. In kidney diseases, the IL-6 levels are effective in predicting mortality risk. To elucidate the relationship between exercise-induced IL-6 elevation, ER stress, and renal physiology, we explored the impact of an acute exhaustive exercise on the ER stress-related proteins and mitochondrial respiratory chain targets in the kidneys of IL-6 knockout (KO) mice. WT and IL-6 KO mice were divided into two subgroups for each phenotype: sedentary (Sed) and 1 h (after 1 h of acute exercise; Ex-1h). The kidneys were removed and prepared for histological, reverse transcription-quantitative polymerase chain reaction (RT-qPCR), and immunoblotting analysis. In summary, IL-6 KO mice had lower degranulated mast cells in the kidney. IL-6 KO mice exhibited reduced exercise performance. The *Hspa5* mRNA levels were significantly increased in response to acute exhaustive exercise in both WT and KO groups, but *Il-10* increased only in response to exercise in the KO group. Additionally, *Ddit3* expression was significantly lower in IL-6 KO mice post-exercise, suggesting a blunted ER stress response without IL-6. At the protein levels, ATF6α expression was notably elevated in IL-6 KO mice following exercise. Regarding mitochondrial protein complexes, we observed lower protein levels of mitochondrial complex IV and CII in the WT Ex-1h group than in the WT Sed. At the same time, the absence of IL-6 did not seem to modify the expression of most mitochondrial complexes in response to acute exercise. Also, publicly available gene expression datasets in humans support our findings, indicating the upregulation of IL-6 signaling and heat shock proteins (HSPs), while decreasing mitochondrial respiratory complex mRNA levels in white blood cells of humans following acute exhaustive exercise. The findings indicate that IL-6 may modulate specific components of ER stress and cytokine responses in the kidney after acute exercise.

## Graphical abstract

**Figure:**
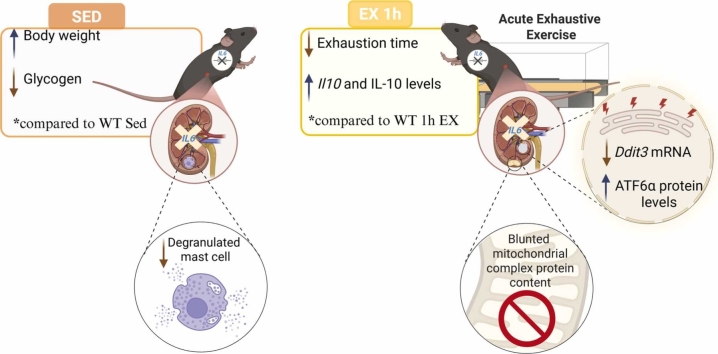
**Graphical Abstract.** Significant results (*P* ≤ .05) were observed in the IL-6 KO SED group compared to the WT SED group (left) and IL-6 KO EX 1 h compared to WT EX 1 h (right).

## Introduction

The kidneys are essential organs for maintaining a range of critical physiological functions, including eliminating metabolic waste and toxins, regulating fluid and electrolyte balance, and preserving the systemic potential of hydrogen homeostasis. Beyond these fundamental roles, these organs also contribute to systemic gluconeogenesis, synthesizing and activating several hormones. Due to their complex and continuous activity, the kidneys are routinely exposed to many environmental and physiological stressors, such as osmotic imbalances, oxidative stress, and endoplasmic reticulum (ER) stress.^1^, ^2^, ^3^

The ER serves as a central mechanism for the entry of numerous secretory and structural proteins, and it is also the primary site for the biosynthesis of cholesterol, steroids, and lipids. It provides a specialized environment that supports proper protein folding, assembly, and disulfide bond formation, all of which are essential for protein synthesis and quality control. ER homeostasis is tightly regulated by an adaptive signaling network known as the unfolded protein response (UPR).^4^, ^5^ This response is mediated by three principal signaling branches, each initiated by distinct ER-resident sensors: activating transcription factor 6 (ATF6), inositol-requiring enzyme 1α, and protein kinase R-like ER kinase (PERK). Under normal conditions, these sensors are inactive due to the association with the endoplasmic reticulum chaperone BIP. However, when misfolded or unfolded proteins accumulate within the ER lumen, BIP dissociates from the sensors to bind these aberrant proteins, thereby triggering UPR activation.^6^, ^7^

The UPR is essential for maintaining normal cellular function, particularly in cells with high protein synthesis rates. Upon activation, UPR supports ER function, promotes recovery from stress, and can confer protection against subsequent cellular insults. However, when ER stress is sustained or unresolved, it can become cytotoxic, ultimately triggering apoptotic pathways. One key mediator of ER stress-induced apoptosis is DNA damage-inducible transcript 3 protein (CHOP; encoded by *Ddit3*), which is upregulated through the PERK–ATF4 signaling axis.^7^, ^8^

ER function is critical for maintaining proteostasis in the kidney, and the disruption of this function, manifested as ER stress, has been implicated in the pathogenesis of several renal disorders. These include primary glomerulonephritis, diabetic nephropathy, acute kidney injury, chronic kidney disease (CKD), and renal fibrosis.^7^ The UPR components are closely integrated with key inflammatory and cellular stress signaling pathways, such as the nuclear factor kappa B (NF-κB), c-Jun N-terminal kinase, and IκB kinase cascades.^9^ The UPR activation has also been associated with increased expression of pro-inflammatory cytokines, including interleukin-6 (IL-6), interleukin-8, and tumor necrosis factor-alpha (TNF-α).^10^

Moreover, IL-6, TNF-α, C-reactive protein, and fibrinogen have been demonstrated to exhibit an inverse relationship with glomerular filtration rate and a direct association with albuminuria.^11^ Cohort studies revealed a direct relationship between high circulating IL-6 levels and reduced kidney function in patients with CKD.^12^, ^13^ In patients with end-stage kidney disease, the plasma IL-6 levels are more effective in predicting mortality risk than IL-1, IL-18, and TNF-α.^14^

IL-6 is a pleotropic target that can exhibit pro- and anti-inflammatory properties.^15^ In response to physical exercise, IL-6 may exert anti-inflammatory effects by modulating other cytokines and signaling pathways.^16^, ^17^ Conversely, excessive or prolonged exercise induces a marked increase in circulating IL-6, which is associated with an inflammatory state.^18^ Regulation of IL-6 levels is essential for maintaining systemic metabolic homeostasis, including controlling glycolysis, fatty acid oxidation, and mitochondrial respiration. Its acute activation supports tissue protection via immune cell recruitment, whereas chronic elevation contributes to persistent and deleterious inflammation.

Intense physical exercise increases plasma concentrations of IL-6, which is also closely linked to muscle metabolism, as it is actively released by contracting skeletal muscle fibers. Pereira et al^19^ reported increased expression of ER stress-related proteins in skeletal muscle following an excessive treadmill running protocol. Pinto et al^20^ also demonstrated that IL-6 knockout (KO) mice exhibited reduced BIP expression in skeletal muscle following an acute exhaustive exercise protocol. Furthermore, ER and mitochondria cooperate to modulate energy metabolism, cellular homeostasis, and maintain survival pathways during exercise.^21^ However, previous studies reported that IL-6 appears to be a factor in exercise-mediated activation of 5'-AMP-activated protein kinase. This cellular energy-sensing target activates transcriptional co-activators (nuclear respiratory factor 1) responsible for longer-term effects such as mitochondrial complex function^22^ and enhanced aerobic capacity.^23^

Although the molecular signatures of IL-6 following exercise have been increasingly investigated, the specific involvement of IL-6 in modulating ER stress signaling and mitochondrial protein complex adaptations in the kidney following acute exhaustive exercise remains largely unexplored. In this study, we compared the renal histological profiles, focusing on inflammation, collagen deposition, and glycogen content, between wild-type (WT) and global IL-6 KO mice for the first time.

To clarify the interplay among exercise-induced IL-6 elevation, ER stress, and renal physiology, this study offers a pioneering analysis of the effects of an acute exhaustive exercise protocol on the expression of ER stress-related proteins and mitochondrial complexes in the kidneys of IL-6 KO mice. Given the established role of IL-6 in inflammation and metabolic regulation during exercise, we hypothesized that IL-6 deficiency would attenuate the ER stress response in renal tissue. Consequently, IL-6 KO mice would display reduced modulation of ER stress markers and a blunted mitochondrial complex protein profile compared to WT controls. These findings aim to advance our understanding of the biological actions of IL-6 in the kidney and to elucidate potential adaptive mechanisms in response to exhaustive exercise. Additionally, we complemented our investigation by analyzing publicly available human transcriptomic data following acute exhaustive exercise to further support the role of IL-6 in exercise-induced responses.

## Materials and methods

### Experimental animals

Eight-week-old C57BL/6 mice from the Central Animal Facility of the Ribeirão Preto campus at the University of São Paulo (USP) were used as the WT group. Global IL-6 KO (IL6^-/-^) mice, on a C57BL/6 background, were obtained from the Laboratory of Molecular Immunology and Embryology at the Transgenose Institute, Centre National de la Recherche Scientifique, and used as the IL-6 knockout (IL-6 KO) group. The mice were housed in micro-insulators (two to three animals per cage) in a ventilated rack (INSIGHT®, Ribeirão Preto, São Paulo, Brazil) with a controlled temperature of 22 ± 2 °C on a 12:12-hour dark-light cycle (light: 6 PM to 6 AM, dark: 6 AM to 6 PM). Food (Purina chow; 63.4% carbohydrate, 25.9% protein, and 10.6% lipids) and water were provided ad libitum. All experimental procedures adhered to the guidelines of the Brazilian College of Animal Experimentation (COBEA) and were approved by the Ethics Committee of the University of São Paulo (I.D. 2018.5.70.90.0). WT and IL-6 KO mice were divided into two subgroups for each phenotype: Sedentary (sedentary; Sed, *n* = 5) and 1 h (after 1 h of acute exercise; Ex-1h, *n* = 5).

### Exercise protocol

The exercise mice (WT and IL-6 KO groups) were acclimated to treadmill running (INSIGHT®, Ribeirão Preto, São Paulo, Brazil) by running for 10 min per day at 6 m·min^−1^ for five consecutive days. Following this adaptation, an incremental load test was conducted, starting at an intensity of 6 m·min^−1^ with a 10-degree incline. The speed was increased by 3 m·min^−1^ every 3 min until exhaustion. This test evaluated the locomotor performance (exhaustion velocity) between the WT and IL-6 KO mice. After 48 h, both groups performed an acute exhaustive exercise protocol on the treadmill at a speed of 20 m·min^−1^ with a 10-degree incline for 90 min. Mice that were exhausted before completing the protocol were withdrawn, and their running times were recorded. The Sedentary group was not subjected to the incremental load test or the acute exercise protocol.

The WT and IL-6 KO mice were euthanized at the following time points: Basal (before the exercise protocol) and after 1 h (1 h) of acute exercise. This acute exhaustive exercise protocol, previously used by Ikeda and colleagues,^24^ demonstrated an increase in IL-6 serum levels 1 h post-exercise compared to baseline. Mice were anesthetized with an intraperitoneal injection of xylazine (10 mg·kg^−1^ of body weight) and ketamine (100 mg·kg^−1^ of body weight). Once the loss of pedal reflexes confirmed the effect of anesthesia, the kidneys were removed.

### Histological analysis

Samples of kidney tissue were previously stored in paraplast for general morphological analysis. Histological Section (5 µm thick) were obtained using a microtome and stained with hematoxylin and eosin (H&E), picrosirius red, periodic acid-Schiff, and toluidine blue. These stained sections were used for analysis and photo documentation at 400x and 200x magnification for each animal in the group. Images were captured from Zeiss using an AxioCam-2 photomicroscope equipped with a digital camera, model AxioCam HR. The tissue volume analysis of the kidney was performed on 12 randomly selected fields at 200x magnification, totaling 60 fields per animal per group, using H&E stain.

#### Mast cell number

The kidney tissue was stained with toluidine blue, and the number of granulated and degranulated mast cells was quantified in 50 fields per animal per group using the cell counter plugin of ImageJ software (version 1.50i) at 400x magnification.^25^

#### Quantification of collagen and glycogen

The amount of collagen was determined using picrosirius red staining, and additional collagen quantification was performed using periodic acid-Schiff staining. These measurements were evaluated based on the intensity of the color. A total of 50 fields per animal per group were analyzed using the IHC-toolbox plugin of ImageJ software (version 1.50i) at 400x magnification.^26^

### Reverse transcription-quantitative polymerase chain reaction

The kidney tissue samples were collected and stored in a −80 ºC freezer until the analysis. All procedures were performed under standard RNase-free conditions to avoid exogenous RNase contamination. Total RNA was isolated using TRIzol®️ Reagent (Thermo Fischer Scientific, Waltham, MA) according to the manufacturer's instructions. Total RNA was quantified by spectrophotometer at OD 260, and quality was checked by the OD 260/280 ratio (BioDrop µLite, Biochrom, Holliston, MA). cDNA was synthesized with 1000 ng of total RNA using a High-Capacity cDNA Reverse Transcription Kit (Applied Biosystems, Foster City, CA). Reverse transcription-quantitative polymerase chain reaction was performed using the StepOne Plus PCR System (Applied Biosystems) to analyze the relative mRNA expression of the target genes (Table 1). *Tbp* was used as a reference gene for the normalization of the data. Relative quantification was calculated by the 2^−ΔΔCT^ method using the Thermo Fisher Cloud Software, RQ version 3.7 (Life Technologies Corporation, Carlsbad, CA, USA).

**Table 1:** The primer's design.

Gene	Forward	Reverse
*Il10*	GCTGGACAACATACTGTAACC	ATTTCCGATAAGGCTTGGCAA
*Xbp1*	GTCCGCAGCACTCAGACTAT	TGCCCAAAAGGATATCAGACTCA
*Hspa5*	GTGTGTGAGACCAGAACCGT	GCAGTCAGGCAGGAGTCTTA
*Ddit3*	ATCTTGAGCCTAACACGTCGAT	GACCAGGTTCTCTCTCCTCAG
*Tbp*	CCTTGTACCCTTCACCAATGAC	ACAGCCAAGATTCACGGTAGA

*Abbreviations: Il10,* Interleukin 10; *Xbp1,* X-box-binding protein 1; *Hspa5,* heat shock protein family A member 5*; Ddit3,* DNA damage-inducible transcript 3; *Tbp,* TATA-box binding protein.

### Immunoblotting technique

The immunoblotting technique was carried out as previously described by our research group.^27^ The antibodies used were Beta-actin (ab213263) and Oxphos (mitochondrial complexes ab110413) from Abcam (Cambridge, UK); BIP (SC-33757) and TLR4 (SC-293072) from Santa Cruz (Santa Cruz Technology, Texas, USA); IL-10 (12163) and ATF6α (65880 s) from Cell Signaling Technology (Cell Signaling Technology, MA, USA). The membranes were stained with Ponceau. All the primary antibodies were utilized at a dilution of 1:1.000, and the secondary antibodies were utilized at a dilution between 1:10.000 to 1:20.000. Images were acquired using the *ChemiDoc*® MP Imaging System (Bio-Rad Laboratories, Hercules, California, USA) and quantified using ImageLab software (Bio-Rad Laboratories). Each protein was normalized to the endogenous control β-actin, detected on the same membrane, except for the mitochondrial complexes (OxPhos), which were normalized to Ponceau staining, as the antibody detects all five complexes across the entire membrane.

### Bioinformatics

To confirm the impact of acute exhaustive exercise on ER stress and mitochondrial respiratory complex, we used a human dataset obtained in the GEO DataSets of the National Institutes of Health (https://www.ncbi.nlm.nih.gov/gds/) accessed on April 6, 2025. Five healthy males performed an exhaustive treadmill test (80% of maximal oxygen uptake/VO_2_max) until individual exhaustion, and blood samples were drawn before and one hour after strenuous exercise (GSE3606). The RNA was isolated from the white blood cells, and gene expression was processed on Affymetrix GeneChips via multiple algorithms using GeneSpring 7.2. We examined Reactome pathways and molecular function enrichment of the top 100 genes upregulated by STRING Interactome 12.0 (Search Tool for the Retrieval of Interacting Genes/Proteins), accessible at https://string-db.org, using a medium confidence score (0.400) and *Homo sapiens* database. Also, cluster heatmap and matrix correlation graphics were created using the SRplot free online platform (https://www.bioinformatics.com.cn/en).

### Statistical analysis

Results are expressed as mean ± standard deviation (SD). Levene's test was used to verify the homogeneity of variances, and the Shapiro-Wilk test was used to check data normality. When normality was confirmed, the unpaired Student's *T* test was used to compare two groups. The two-way analysis of variance (ANOVA) was used to compare factor 1: genotype, and factor 2: condition (sed versus ex), followed by a Tukey's post hoc test when the two-way ANOVA indicated significance. All statistical analyses were set at *P*<.05 and two-sided. Statistical analyses were performed using GraphPad Prism v.8.0.1 for Windows (GraphPad Software, CA, USA).

## Results

### The IL6 absence decreases the degranulated mast cells in the kidney

**Fig. 1:**
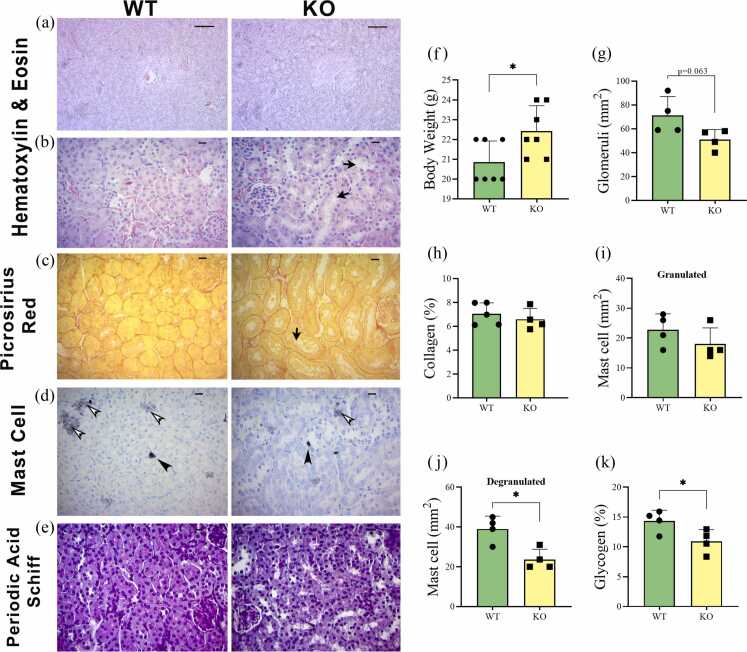
*Histological characterization of kidney tissue in WT and IL-6 KO mice.***(A-B)** Histological characterization of kidney tissue by hematoxylin-eosin (H&E) (A - x200 magnification) (B - x400 magnification) (arrow: tubular lumen - tubular dilatation). **(C)** Collagen by picrosirius red (x400 magnification) (arrow: tubular lumen - tubular dilatation). **(D)** Mast cells by toluidine blue (x400 magnification) (black arrowhead: granulated mast cells; white arrowhead: mast cell degranulation). **(E)** Glycogen by periodic acid Schiff (PAS) (x400 magnification). **(F)** Body weight. **(G)** Number of glomeruli (mm^2^). **(H)** Percentage of collagen. **(I)** Number of the mast cell granulated (mm^2^). **(J)** Number of the mast cell – cell-degranulated (mm^2^). **(K)** Percentage of glycogen. Data are presented as mean ± SD (*n* = 7 to 4 mice per group). *P ≤ .05.

Several histological staining techniques were adopted to show the kidneys' histoarchitecture (Figure 1A-E), in which the number of glomeruli (Figure 1G) was reduced, but without a statistical difference (*P* = .063). The body weight of IL-6 KO mice was higher (*P* < .05) than that of WT mice (Figure 1F). No significant differences were observed between groups in kidney collagen deposition or the granulated mast cells (Figures 1H and I). The absence of IL-6 impaired the inflammatory response, as evidenced by a significant reduction in degranulated mast cells in KO mice compared to WT mice (Figure 1J). For renal intracellular glycogen quantification (Figure 1K), a significant reduction was observed in the KO group compared to the WT group, further indicating metabolic alterations associated with IL-6 deficiency.

### The exercise increases ATF6α protein in IL6KO mice

**Fig. 2:**
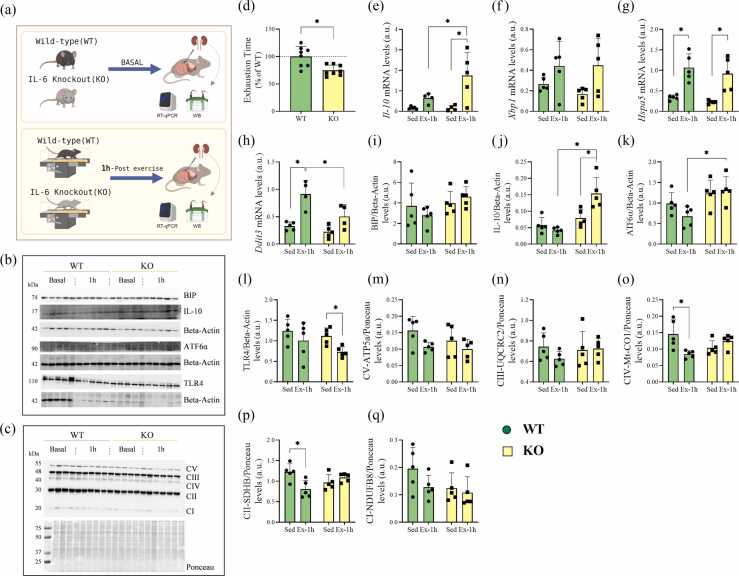
*Acute exercise modulates gene expression and protein levels of endoplasmic reticulum stress and mitochondrial markers in the kidney.***(A)** Experimental Design. **(B-C)** Representative membranes. **(D)** Exhaustion time (% of WT). **(E)***Il-10* mRNA levels (a.u.). **(F)***Xbp1* mRNA levels (a.u.). **(G)***Hspa5* mRNA levels (a.u.). **(H)***Ddit3* mRNA levels (a.u.). **(I)** BIP levels (a.u). **(J)** IL-10 levels (a.u.). **(K)** ATF6α levels (a.u). **(L)** TLR4 levels (a.u.). **(M)** CV-ATP5a levels (a.u). **(N)** CIII-UQCRC2 levels (a.u). **(O)** CIV-Mt-CO1 levels (a.u). **(P)** CII-SDHB levels (a.u). **(Q)** CI-NDUFB8 levels (a.u). The protein values were divided by the Ponceau. Data are presented as mean ± SD (*n* = 7-5 mice per group). *P ≤ .05. a.u. = Arbitrary units.

The IL-6 KO group had a lower exhaustion time on the incremental load test than the WT group (Figure 2D). The mRNA levels of *Il-10* (Figure 2E) were higher for the IL-6 KO Ex-1h than the IL-6 KO Sed and WT Ex-1h. The mRNA levels of *Xbp1* (Figure 2F) were not different between the experimental groups. The levels of *Hspa5* (Figure 2G) were higher for both exercise groups (WT Ex-1h and IL-6 KO Ex-1h) than the Sed groups. The levels of *Ddit3* (Figure 2H) were higher for the WT Ex-1h group than the WT Sed and IL-6 KO Ex-1h groups. The protein contents of BIP, TLR4, mitochondrial complex V (ATP5a), III (UQCRC2), and I (NDUFB8) were not different between the experimental groups (Figures 2I and L-N, respectively). The protein content of IL-10 (Figure 2J) was higher for the IL-6 KO Ex-1h compared to IL-6 KO Sed and WT Ex-1h. The protein content of ATF6α (Figure 2K) was higher for the IL-6 KO Ex-1h group than the WT Ex-1h group. The protein levels of mitochondrial complex IV (Mt-CO1) and CII (SDHB) (Figure 2O-P) were lower for the WT Ex-1h compared to WT Sed. The representative membranes are demonstrated in Figure 2B and C.

### Bioinformatic analysis reveals increased expression of heat shock proteins (HSPs) and decreased mitochondrial respiratory complex mRNA levels in humans following acute exhaustive exercise

**Fig. 3:**
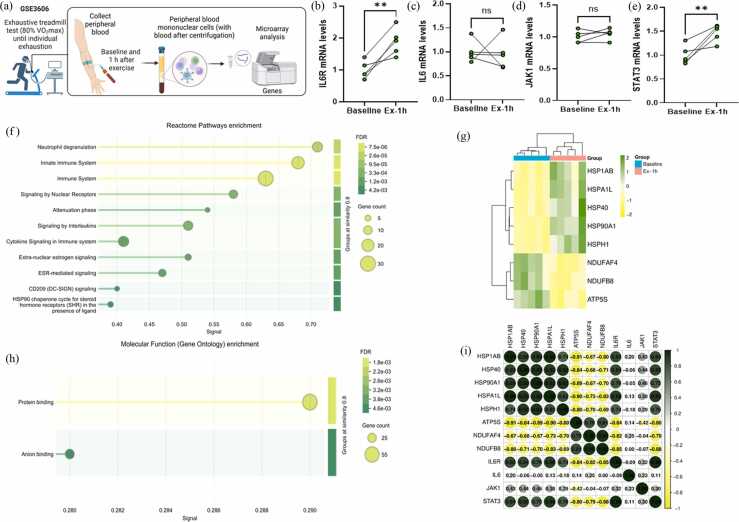
*Bioinformatics of heat shock proteins (HSPs) and mitochondrial respiratory genes in the PBMCs of humans following acute exhaustive exercise.***(A)** experimental design (Series GSE3606), **(B)** IL6R mRNA levels, **(C)** IL6 mRNA levels, **(D)** JAK1 mRNA levels, **(E)** STAT3 mRNA levels, **(F)** Reactome Pathways enrichment of top 100 upregulated genes, **(G)** heatmap of HSPs and mitochondrial genes (top 100 genes regulated), **(H)** molecular function of top 100 upregulated genes, **(I)** matrix correlation.

**Fig. 4:**
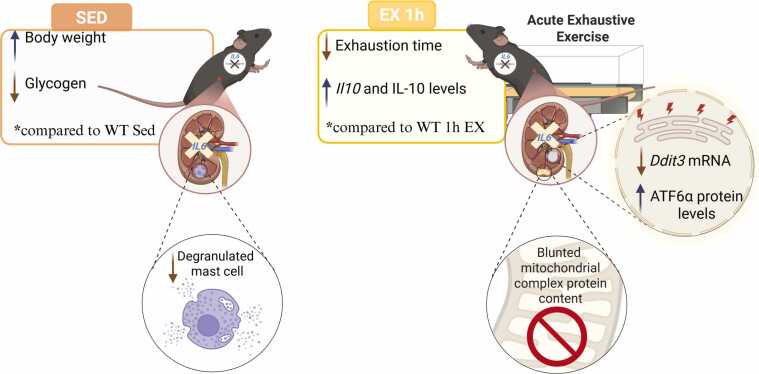
*Graphical Abstract.* Significant results (*P* ≤ .05) were observed in the IL-6 KO SED group compared to the WT SED group (left) and IL-6 KO EX 1 h compared to WT EX 1 h (right).

Excessive exercise induced elevated mRNA levels of *IL6R* (*P* = .0043; Figure 3A) and *STAT3* (*P* = 0.0046; Figure 4E) in peripheral blood mononuclear cells (PBMCs) from healthy individuals 1 h post-exercise. No significant changes were observed in *IL6* (Figure 3C) or *JAK1* (Figure 3D) expression between time points (*P* > .05). Gene Ontology enrichment analysis of the top 100 upregulated genes at 1-h post-exercise revealed enrichment in the chaperone cycle (FDR = 0.0423) and cytokine signaling in the immune system (FDR = 0.0147) (Figure 3F). These genes were also associated with the molecular function of protein binding (FDR = 0.0187), suggesting acute activation of the ER stress response (Figure 3H).

The heatmap illustrates increased expression of specific HSPs, including *HSP1AB*, *DNAJB1* (HSP40), *HSP90AA1*, *HSPA1L*, and *HSPH1,* following exercise, along with reduced expression of mitochondrial respiratory chain genes such as *ATP5S*, *NDUFAF4*, and *NDUFB8* (Figure 3G). Notably, *IL6R* and *STAT3* expression levels showed a strong positive correlation with *HSP1AB*, *DNAJB1*, and *HSPA1L*, and a negative correlation with genes involved in mitochondrial function (*P* < .05; Figure 3I).

## Discussion

The main findings of the present investigation were that IL-6 KO mice had lower degranulated mast cells in the kidney; the IL-6 KO group showed significantly reduced exhaustion time compared to WT mice, which indicates impaired exercise performance in the absence of IL-6; *Il10* increased substantially after exercise in the KO group, suggesting that IL-6 deficiency may enhance *Il10* transcription in the kidney; *Ddit3* was induced by exercise in WT mice, but this response was attenuated in IL-6 KO mice, suggesting a reduced pro-apoptotic ER stress signaling (CHOP) when IL-6 is absent; ATF6α showed higher levels in IL-6 KO mice post-exercise, indicating possible compensatory upregulation in the absence of IL-6, and the IL-6 KO mice did not show modulation of the mitochondrial complexes. Additionally, publicly available gene expression data revealed activation of IL-6 signaling and increased mRNA levels of HSPs in the PBMCs of healthy humans following acute exhaustive exercise.

Regardless of the underlying cause, CKD is characterized by a sustained inflammatory response, marked by the production of pro-inflammatory mediators and infiltration of immune cells into the tubulointerstitial space. Among these immune cells, mast cells play a notable role. These granulated cells secrete a broad range of inflammatory mediators, such as histamine, serotonin, cytokines, chemokines, and proteases, which are rapidly released upon activation via specific cell surface receptors.^28^, ^29^

Normal kidney parenchyma contains only a small number of mast cells (MCs); however, in many renal pathologies, their presence is significantly elevated compared to healthy kidneys.^30^ This increase frequently correlates with higher serum creatinine levels and is associated with the severity of tubulointerstitial injury and fibrosis.^30^, ^31^ The observation in the present study that IL-6 KO mice exhibited fewer degranulated MCs in the kidney while maintaining a normal number of granulated (non-activated) MCs suggests that IL-6 may be involved in MC activation rather than recruitment in this tissue.

The kidney not only reabsorbs filtered glucose but also produces it through gluconeogenesis. Glycogen, a highly branched glucose polymer, acts as a storage form that can be mobilized when needed.^32^ Under normal conditions, renal glycogen levels are temporary and influenced by fasting,^32^ with insulin suppressing both glucose production and release.^33^ Unlike the pathological glycogen buildup seen in diabetes,^34^, ^35^ IL-6 KO mice in our study showed lower renal glycogen content than WT littermates.

Although IL-6 is known to regulate glucose and glycogen metabolism in skeletal muscle and liver, its role in renal glycogen metabolism remains unclear. No direct evidence currently links IL-6 to glycogen dynamics in the kidney. It is plausible that IL-6 influences renal glycogen under pathological conditions; however, further studies are needed to clarify the mechanisms and establish causality.

Several studies have shown that IL-6 KO mice exhibit reduced exercise tolerance.^36^, ^37^, ^38^, ^39^, ^40^ For example, Wojewoda et al^37^ and Pinto et al^20^ reported poor exercise performance in IL-6 KO mice, which is consistent with the present findings. IL-6 appears to play a role in maintaining energy homeostasis during physical activity,^40^ supporting the hypothesis that IL-6 may act as an energy sensor in response to exercise demands.

Also, it is essential to highlight that our study did not address biological sex differences, which limits generalizability given the influence of sex on immune, metabolic, and renal responses to exercise. Females have been reported to exhibit superior IL-6 responses than males following stress-inducing protocols.^41^ In addition, estradiol can exert renoprotective effects, enhancing filtration and reducing injury biomarkers.^42^ Other studies demonstrated that estrogen could attenuate inflammation, including IL-6 signaling.^43^, ^44^ Therefore, future studies should include sex-specific analyses to clarify how sex influences IL-6-driven ER stress responses in the kidney following exercise.

The research on the impact of IL-6 deficiency on renal metabolism is limited, with even fewer investigations addressing the correlation between the absence of IL-6 and ER stress in the kidney post-exercise, highlighting the novelty of the present study. To the best of our knowledge, no investigations have specifically examined ER stress responses in the kidneys of IL-6 KO mice.

In the presence of kidney diseases, a recent study reported that X-box-binding protein1 (XBP1)-KO mice exhibited podocyte injury and affected UPR.^45^ Rats with diabetes mellitus showed higher levels of GRP78, p-PERK, ATF4, and CHOP in the kidneys, as well as enhanced apoptosis.^46^ Scientific literature firmly establishes that both acute and CKDs are linked to ER stress and the activation of UPR pathways.^47^ Additionally, elevated levels of serum pro-inflammatory cytokines, such as IL-6, have been identified as potential predictors of CKD onset and progression.^3^, ^47^

Contrary to expectations, the WT mice displayed lower protein levels of mitochondrial complexes IV and II after the acute exercise in the kidney. Using a diabetic mouse model, Liu et al^48^ showed that exercise training significantly increased citrate synthase, mitochondrial complexes I, II, and V, and PGC1α at the protein level in the kidney of *db/db* + Ex mice compared with non-exercise *db/db* mice. In type 2 diabetes, an increase in renal gluconeogenesis and glucose uptake has been observed, suggesting that the kidney demands greater mitochondrial activity to meet the elevated energy requirements associated with altered glucose metabolism. In this context, the reduced glycogen levels detected in the kidneys of IL-6 KO mice may reflect a compensatory metabolic adaptation. This observation could be linked to the absence of significant changes in the expression of mitochondrial respiratory chain complexes, indicating that IL-6 deficiency may limit the kidney's capacity to adjust mitochondrial function despite altered energy substrate availability.

Animal studies have shown that acute exhaustive exercise increases reactive oxygen species production in skeletal muscle while reducing mitochondrial respiratory complex protein levels via inhibition of the PGC-1α-Nrf1/Nrf2-TFAM pathway.^49^ In the present study, we observed decreased protein levels of mitochondrial complexes IV and II in the WT Ex-1h group compared to WT Sed, supporting the reduction in mitochondrial respiratory complex mRNA levels observed in human datasets following acute exhaustive exercise. In contrast, IL-6 KO mice showed no changes in renal mitochondrial complex expression, supporting the role of IL-6 as a key mediator of mitochondrial metabolism during acute exercise. This effect may involve acute regulation of energy-sensing pathways such as 5'-AMP-activated protein kinase and transcription factors like PPARα, which modulate mitochondrial function during exercise.^50^

Using this same animal model, Pinto et al^20^ demonstrated modulation of ER stress proteins in the skeletal muscle in response to an acute exercise protocol.^20^ In contrast to the skeletal muscle findings, BIP expression in the kidney did not differ between groups; however, expression of *Ddit3* (the gene encoding CHOP) was attenuated following exercise in IL-6 KO mice, while ATF6α expression was upregulated, which could indicate reduced pro-apoptotic signaling, followed by a compensatory upregulation for the IL-6 absence.

PBMCs are a useful surrogate material for assessing protein or gene expression patterns that reflect those in other tissues, which are not easily accessible through human biopsies. Using bioinformatics tools, we discovered higher HSP mRNA levels in the PBMCs of humans following acute exhaustive exercise. Moreover, IL6R and STAT3 exhibited a strong positive correlation with several chaperones, supporting the hypothesis that IL-6 signaling can mediate ER activation and UPR pathways during strenuous exercise.

In conclusion, we hypothesized that IL-6 may be necessary for the full activation of this ER stress-related apoptotic pathway in response to acute exercise in the kidney of male mice. Regarding mitochondrial protein content, the absence of IL-6 did not significantly alter the expression of respiratory chain complexes after acute exhaustive exercise. Longitudinal or repeated-measures designs would be essential to determine whether the observed changes in the present study reflect transient stress, adaptive remodeling, or pathological alterations. Future studies should incorporate multiple time points and/or chronic exercise interventions to clarify the temporal dynamics and long-term implications of IL-6 signaling in the kidney. Figure 4 shows the results observed in the IL-6 KO mice compared to the WT mice.

## Study limitations

It is essential to acknowledge that a limitation of the present study is the absence of biochemical analyses, including measurements of serum creatinine, urea, and albumin, as well as renal function, in male and female mice.

## Funding and support

The authors acknowledge the financial support from the São Paulo Research Foundation (FAPESP; process numbers 2019/11820-5, 2021/06291-3, 2021/08693-1, 2021/14233-3, 2022/09100-7, and 2022/15670-0), the National Council for Scientific and Technological Development (CNPq; process numbers 308999/2022-3; 303766/2022-0), and the Coordination for the Improvement of Higher Education Personnel (CAPES; Finance Code 001).

## CRediT authorship contribution statement

**Adelino S.R. da Silva:** Writing – review & editing, Writing – original draft, Visualization, Supervision, Funding acquisition, Data curation, Conceptualization. **Caroline M. da Luz:** Writing – review & editing, Visualization, Methodology, Data curation. **Bruno B. Marafon:** Writing – review & editing, Visualization, Methodology. **Maria Eduarda A. Tavares:** Writing – review & editing, Visualization, Methodology. **Ivo Vieira de S. Neto:** Writing – review & editing, Visualization, Methodology. **Ruither O. Gomes Carolino:** Writing – review & editing, Visualization, Methodology. **Driele C. da Silva Ferreira:** Visualization, Methodology. **Julia T. Marinho:** Visualization, Methodology. **Giovana R. Teixeira:** Writing – review & editing, Visualization, Methodology, Funding acquisition. **Dennys E. Cintra:** Writing – review & editing, Visualization, Funding acquisition. **José R. Pauli:** Writing – review & editing, Visualization, Funding acquisition. **Eduardo R. Ropelle:** Writing – review & editing, Visualization, Funding acquisition. **Ellen C. de Freitas:** Writing – review & editing, Visualization, Funding acquisition. **Ana P. Pinto:** Writing – review & editing, Writing – original draft, Visualization, Supervision, Methodology, Data curation, Conceptualization.

## Declaration of Generative AI and AI-Assisted Technologies in the Writing Process

During the preparation of this study, the authors (s) used ChatGPT to improve English grammar and readability. After using this tool/service, the author(s) reviewed and edited the content as needed and took (s) full responsibility for the content of the publication.

## Declaration of Competing Interest

The authors declare the following financial interests/personal relationships, which may be considered as potential competing interests: Ana Paula Pinto reports financial support was provided by the State of Sao Paulo Research Foundation. Ana Paula reports a relationship with the State of Sao Paulo Research Foundation that includes: funding grants. Adelino Sanchez Ramos da Silva reports a relationship with the State of Sao Paulo Research Foundation that includes: funding grants. Caroline M. da Luz reports a relationship with State of Sao Paulo Research Foundation that includes: funding grants. Bruno B. Marafon reports a relationship with the State of Sao Paulo Research Foundation that includes: funding grants. Ivo Vieira de S. Neto reports a relationship with the State of Sao Paulo Research Foundation that includes: funding grants. If there are other authors, they declare that they have no known competing financial interests or personal relationships that could have appeared to influence the work reported in this paper.

## Data Availability

Data will be made available on request.
